# A review of clinical use of surface-enhanced Raman scattering-based biosensing for glioma

**DOI:** 10.3389/fneur.2024.1287213

**Published:** 2024-04-08

**Authors:** Guohui Yang, Kaizhi Zhang, Weiqing Xu, Shuping Xu

**Affiliations:** ^1^China-Japan Union Hospital of Jilin University, Changchun, China; ^2^State Key Laboratory of Supramolecular Structure and Materials, College of Chemistry, Jilin University, Changchun, China; ^3^Institute of Theoretical Chemistry, College of Chemistry, Jilin University, Changchun, China; ^4^Center for Supramolecular Chemical Biology, College of Chemistry, Jilin University, Changchun, China

**Keywords:** SERS, glioma, early diagnosis, SERS tag, boundary

## Abstract

Glioma is the most common malignant tumor of the nervous system in recent centuries, and the incidence rate of glioma is increasing year by year. Its invasive growth and malignant biological behaviors make it one of the most challenging malignant tumors. Maximizing the resection range (EOR) while minimizing the impact on normal brain tissue is crucial for patient prognosis. Changes in metabolites produced by tumor cells and their microenvironments might be important indicators. As a powerful spectroscopic technique, surface-enhanced Raman scattering (SERS) has many advantages, including ultra-high sensitivity, high specificity, and non-invasive features, which allow SERS technology to be widely applied in biomedicine, especially in the differential diagnosis of malignant tumor tissues. This review first introduced the clinical use of responsive SERS probes. Next, the sensing mechanisms of microenvironment-responsive SERS probes were summarized. Finally, the biomedical applications of these responsive SERS probes were listed in four sections, detecting tumor boundaries due to the changes of pH-responsive SERS probes, SERS probes to guide tumor resection, SERS for liquid biopsy to achieve early diagnosis of tumors, and the application of free-label SERS technology to detect fresh glioma specimens. Finally, the challenges and prospects of responsive SERS detections were summarized for clinical use.

## 1 Introduction

**Figure 1:**
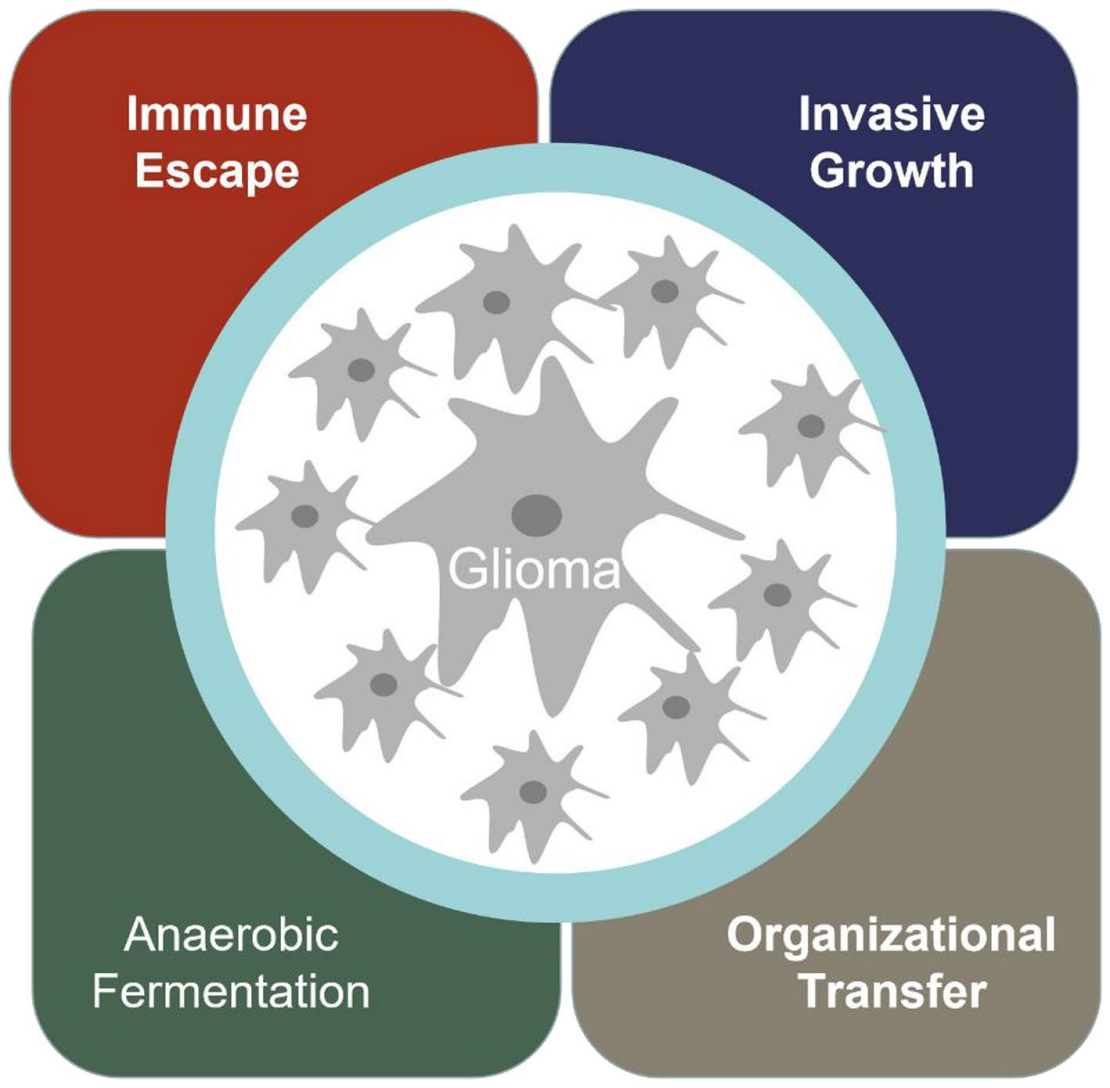
The hallmarks of glioma tumor.

Cancer has become the most significant disease that troubles human society. Glioma is one of the most common malignant tumors in the nervous system in the world, and the incidence rate is increasing year by year (1). When normal cells transform into malignant tumor cells, they acquire special abilities, such as immune escape, infinite proliferation, invasive growth, anaerobic digestion, and promoting vascular proliferation (Figure 1).

In the process of tumor development, changes in metabolites produced by tumor cells and their microenvironments often precede variation in their imaging, and also play a certain guiding role in the developing mechanisms of tumor cells. For example, glioma cells have the common value-added characteristics of general malignant cells, which can be glycolytic in an oxygen-free environment, so that the local microenvironment of tumor cells becomes acidic. At the same time, intracellular matrix metalloenzyme, one of the main reasons for glioma cells causing invasive damage, and the elevated cytokine interleukin-1 in Glioma β (IL-1 β) and tumor necrosis factor-α (TNF-α), are all overexpressed (2). Therefore, the proliferation, migration, and invasion of cancer cells are accompanied by significant changes in tumor-related metabolites and microenvironments (3, 4). Monitoring the metabolites and microenvironment of tumor tissue can serve as a primary method for diagnosing and treating cancer, which has received widespread attention in biomedical applications.

At present, surgical resection is still the main way to treat malignant tumors. Due to the invasive nature of glioma, the main problem of glioma surgery is to retain normal brain tissue while resecting glioma tissue as much as possible. It is of great significance for prolonging the survival period and improving the quality of life of patients, and can minimize the occurrence of postoperative complications to the greatest extent possible (5). However, due to the invasive nature of malignant tumors, identifying the boundaries of malignant tumors is particularly difficult. Many imaging technologies have been used for guiding the diagnosis and treatment of glioma, determining the boundaries of gliomas in clinical practice, for instant, pre-operative and intraoperative magnetic resonance imaging (MRI), fluorescence, intraoperative ultrasound, and intraoperative neuroelectrophysiological testing. Pre-operative nuclear magnetic plain scan and enhanced examination can effectively locate and qualitatively identify the location of tumors and accurately identify the relationship between functional areas. However, there are significant differences in functional and anatomical aspects. The variability between individuals, the impact of brain tumors, and their associated mass effects may distort common anatomical markers, making anatomical-based functional localization inaccurate (6). In recent years, intraoperative MRI and intraoperative ultrasound have been widely used to resect gliomas (7). It has been fully demonstrated that using intraoperative MRI significantly improved the surgical success rate and prognosis of glioma patients. However, it cannot be denied that intraoperative MRI and intraoperative ultrasound imaging have some limitations. Artifacts often appear in intraoperative ultrasound imaging, and intraoperative MRI, which needs to terminate the surgical process and is a major challenge for surgeon (7). Fluorescence imaging related to tumor metabolites has been widely used in clinical practice in recent years (8), especially the dyes with an emission in the near-infrared spectroscopic range (9). So far, the clinically allowed auxiliary imaging agents mainly include fluorescein sodium (FLS), indocyanine green (ICG), and 5-amino Levulinic acid (5-ALA) (10–12). Under normal conditions, fluorescein sodium has a large molecular weight and cannot penetrate the normal blood–brain barrier. However, due to the invasive growth of glioma cells, vascular endothelial cells were damaged so that fluorescein could enter tumor tissue through the blood–brain barrier, giving unique yellow-green fluorescence. However, the inherent drawbacks of fluorescence, such as rapid bleaching and short blood circulation, hinder clinical development (13, 14). Intraoperative neuroelectrophysiological monitoring technology is another technique that mainly focuses on the removal of gliomas located in the functional area. It can effectively avoid damaging the main functional nerves during the removal of gliomas located in the functional area, while preserving some neural function while maximizing tumor resection. However, intraoperative electrophysiological monitoring cannot identify the boundaries of gliomas (15). Among the commonly used techniques nowadays, an examination technique that can quickly, sensitively, and accurately determine the boundaries of gliomas is still highly required. The advantages and disadvantages of these technologies are summarized in Table 1.

**Table 1:** Advantages and disadvantages of glioma detection technology.

Timepoint	Technique	Advantages	Disadvantage
Preoperative	Magnetic resonance imaging (MRI)	Locate and qualitatively identify the location of tumors and accurately identify the relationship between functional areas.	There are significant differences in functional and anatomical aspects. The variability between individuals, the impact of brain tumors, and their associated mass effects may distort common anatomical markers, making anatomical-based functional localization inaccurate
Intraoperative	Magnetic resonance imaging (MRI)	Avoiding anatomical displacement caused by tissue traction in preoperative nuclear magnetic resonance imaging	Surgical process needs to be terminated
Intraoperative	Fluorescence imaging	Fluorescein could enter tumor tissue through the blood–brain barrier, giving unique yellow-green fluorescence. Therefore, it can be used to detect tumor boundaries	(1) Rapid bleaching and short blood circulation. (2) Neurotoxicity
Intraoperative	Neuroelectrophysiological monitoring	It can effectively avoid damaging the main functional nerves during the removal of gliomas located in the functional area, while preserving some neural function while maximizing tumor resection.	It cannot identify the boundaries of gliomas
Intraoperative	surface-enhanced Raman scattering (SERS)	(1) High specificity:the SERS spectrum reflects the intrinsic characteristic structures of different molecules in the form of fingerprints. (2) High sensitivity: High signal strength. (3) *In situ* detection. This technology can achieve *in situ* detection, which means that molecules can be measured from their original positions, no matter tumor cell tissues, its microenvironments, or interstitial fluids. (4) No interference from water	There are errors in the spectral acquisition process

Raman scattering originates from the inelastic scattering of light, which can directly reflect the vibration/rotational vibration information in molecules and materials (16). Due to the specific spectral effects of Raman scattering on specific biological molecules, it can be used for imaging tissues and cells (17). In addition, Raman scattering has minimal sample preparation, low water molecule interference, and the ability to simultaneously monitor multiple molecules, making it an ideal method for detecting tumor-related metabolites (18). However, normal Raman scattering is usually a very weak process since only one out of approximately 10^8^ photons will spontaneously undergo one Raman scattering photon (19). This inherent weakness limits the strength of the available Raman signal. It is found that molecules adsorbed on rough precious noble metal surfaces can realize a significantly enhanced Raman signal by a billion orders of magnitude (20), noted as surface-enhanced Raman scattering (SERS) (21). SERS overcomes the shortcomings of weak Raman scattering signals, making SERS an applicable tool for biomedical applications. SERS mainly has the following advantages for biomedical purposes: (1) High specificity. Due to the different spectral characteristics of SERS generated by different molecules, the SERS spectrum reflects the intrinsic characteristic structures of different molecules in the form of fingerprints, thus possessing the advantages of high specificity. (2) High sensitivity. Molecules adsorbed on rough precious metal surfaces can enhance a Raman signal by a fold of about 10^6^, contributing to subtle spectral change extraction (21). (3) *In situ* detection. This technology can achieve *in situ* detection, which means that molecules can be measured from their original positions, no matter tumor cell tissues, its microenvironments, or interstitial fluids (22, 23). (4) No interference from water. Since water can provide signals in many spectral ranges (infrared, terahertz and microwave, etc), it will be troubled when applying those methods in water systems. For Raman and SERS approaches, most tissues and cells give signals in a range of 400–2,000 cm^−1^, which has no overlaps with water. SERS technology has already become a promising detection method for biomedical testing, liquid biopsy, and *in vitro* diagnosis (IVD) (24, 25).

In this review, we focus on SERS applications in glioma-related systems. First, the SERS nanoprobes were introduced, followed by a summary of their configurations and responsive mechanisms. Next, the biomedical applications of these responsive SERS probes are listed and presented in detail: (1) detecting tumor boundaries using pH-responsive SERS probe, (2) using SERS-active nanoparticles to guide tumor resection, (3) SERS technology for liquid biopsy for early diagnosis of tumors, and (4) free-label SERS technology of fresh specimens for qualitative diagnosis of glioma. Finally, we will state the challenges of SERS in the clinic fields and prospects for future progress.

## 2 SERS probes for clinical use

**Figure 2:**
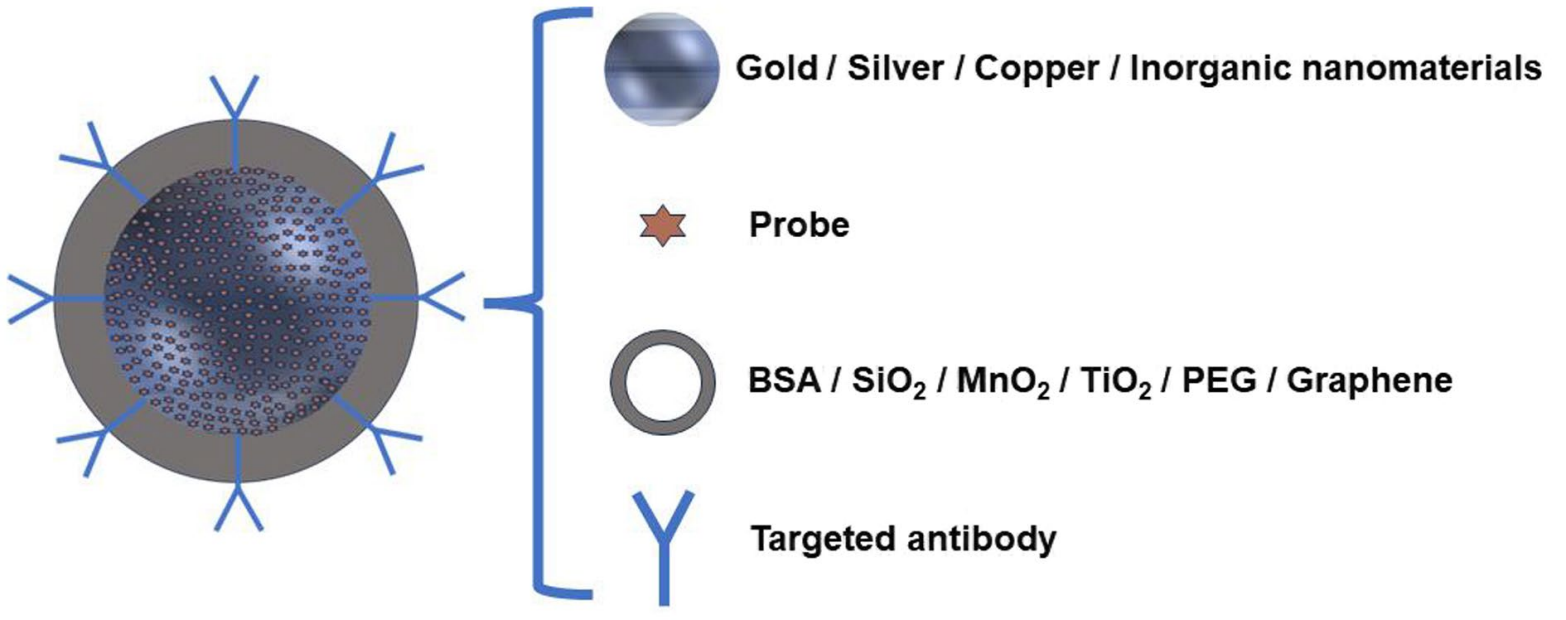
Schematic illustration of the immuno-SERS nanoprobe.

SERS is strongly dependent on SERS substrates. As we all know, SERS substrates are mainly represented in two forms, solid-supporting substrates and colloidal nanoparticles. Colloidal SERS-active nanoparticles are dominantly used in biomedical fields. In most sensing strategies, indirect SERS was adopted, and SERS tags with a noble metal nanoparticle decorated with Raman reporter molecules (sometimes a protective layer is also needed) were fabricated. Plasmonic nanomaterials including gold and silver are the first choices of most SERS clinical studies. Very limited publications utilized the SERS-active semiconductor materials, e.g., metal oxides, silver halide, single-element semiconductors, and semiconductor sulfides/arsenides (26). The materials of plasmonic nanoparticles have significant impacts on the SERS intensities. Gold nanospheres, nanorods, and nanostars are highly stable and not easily oxidized, which have been chosen in many studies. Although silver nanoparticles are prone to oxidation, the Raman signals generated above Ag exhibit much stronger than that of gold nanoparticles (27). Optimization of size and shape allows passive enrichment of nanoparticles to the tumor location. Nanoprobes with specific sizes can pass through tumor tissue but not normal tissue (28), which can be explained by the permeability enhancement effect that is caused by the destruction of blood vessels around the tumor tissue and the retention effect due to the destruction of the lymphatic channels around the tumor tissue, reducing the reflux of the nanoprobe. Thus, the edge of the tumor can be delineated and depicted according to the residue of the nanoprobe. To track immune information on tissue samples, the immuno-SERS tags (Figure 2) were employed (29), which can provide feedback on immune information on the surface of tissue samples, similar to immunohistochemistry. These SERS tags were decorated with antibodies to enrich them with high-specific identification functions, and they use the fingerprint characteristics of reporter molecules to simultaneously realize multiplex detections of antigens and targets.

A highly sensitive and responsive SERS nanoprobe is preferred, which can respond quickly to tumor microenvironments that become an indicator for malignant lesions. A stable, responsive SERS probe typically consists of three parts: a noble metal substrate, responsive Raman reporter molecules, and a protective layer (30). A typical pH-responsive probe is fabricated by attaching pH-sensitive Raman reporters to plasmonic nanoparticles. With the change of pH value in the environment, the reporter molecule will undergo structural changes due to the protonation/deprotonation, and its vibration mode will also undergo corresponding changes (31). Thus, different Raman signals infer different pHs, pointing to the intracellular microenvironments (32). The commonly used pH-responsive Raman reporters include 4-mercaptopyridine (4-MPY), *p*-aminophenylthiol (p-ATP), 3-amino-5-mercapto-1, 2, 4-triazole (AMT), and 2-aminophenylthiol (2-ABT), etc. (33–39).

In addition, to avoid unspecific binding from the other matrices, a protective layer is needed to protect them from damage and replacement. At present, the protective layers include bovine serum albumin (BSA), SiO_2_, MnO_2_, TiO_2_, and organic polymers (40, 41). Sometimes, organic polymers (pegylated) (42) or carbon (graphitic) shells (43) are also used. Nowadays, SiO_2_ is a common protective layer, which is usually formed by the decomposition of Na_2_SiO_3_ or tetraethyl orthosilicic acid (44, 45). The protective layer can also prevent SERS tags from the influence of working surroundings. For example, a metal–organic framework (MOF) as a shell can protect Au nanoparticles from aggregation, which was used for indicating tumor edge under SERS imaging (46).

Endowing the specific target recognition feature to SERS tags for the cell membrane and organelle surface has been well designed in recent years. Kircher et al. (47) injected Au@SiO_2_ nanoprobes coated with 1, 4, 7, 10-tetraazacyclododecane-1, 4, 7, 10-tetraacetic acid (DOTA) into the tail vein of mice for SERS imaging. The aggregation of nanoprobes in tumor tissue can clearly observe the tumor area, thus revealing tumor tissue that cannot be detected by the naked eye. In addition, Vendrell et al. (48) developed an efficient tumor-targeting nanostructure based on the single-walled carbon nanotubes (SWNTs), which provides a strong and fixed Raman peak at 1,593 cm^−1^. SWNTs were decorated with an RGD peptide (arginyl-glycyl-aspartic acid) to increase the cancer cell internalization efficiency. This nanoprobe was injected through the tail vein of mice to identify boundaries by tracking the location of probes.

## 3 Application of responsive SERS probes in biomedical fields

### 3.1 Tumor cell microenvironments revealed by SERS

The metabolic growth and development of cells are often accompanied by the acidification of extracellular fluid, which is often accompanied by cell aging, apoptosis, proliferation, etc., especially for tumor cells. The tumor’s extracellular fluid is often accompanied by a change in extracellular pH (49). Therefore, detecting the pH of extracellular fluids to be acidic can be a sign of tumors. Research shows that the acidification of extracellular fluid is often related to the invasion of nausea tumors. Therefore, exploring a pH-responsive SERS probe becomes a feasible way to distinguish tumor boundaries.

**Figure 3:**
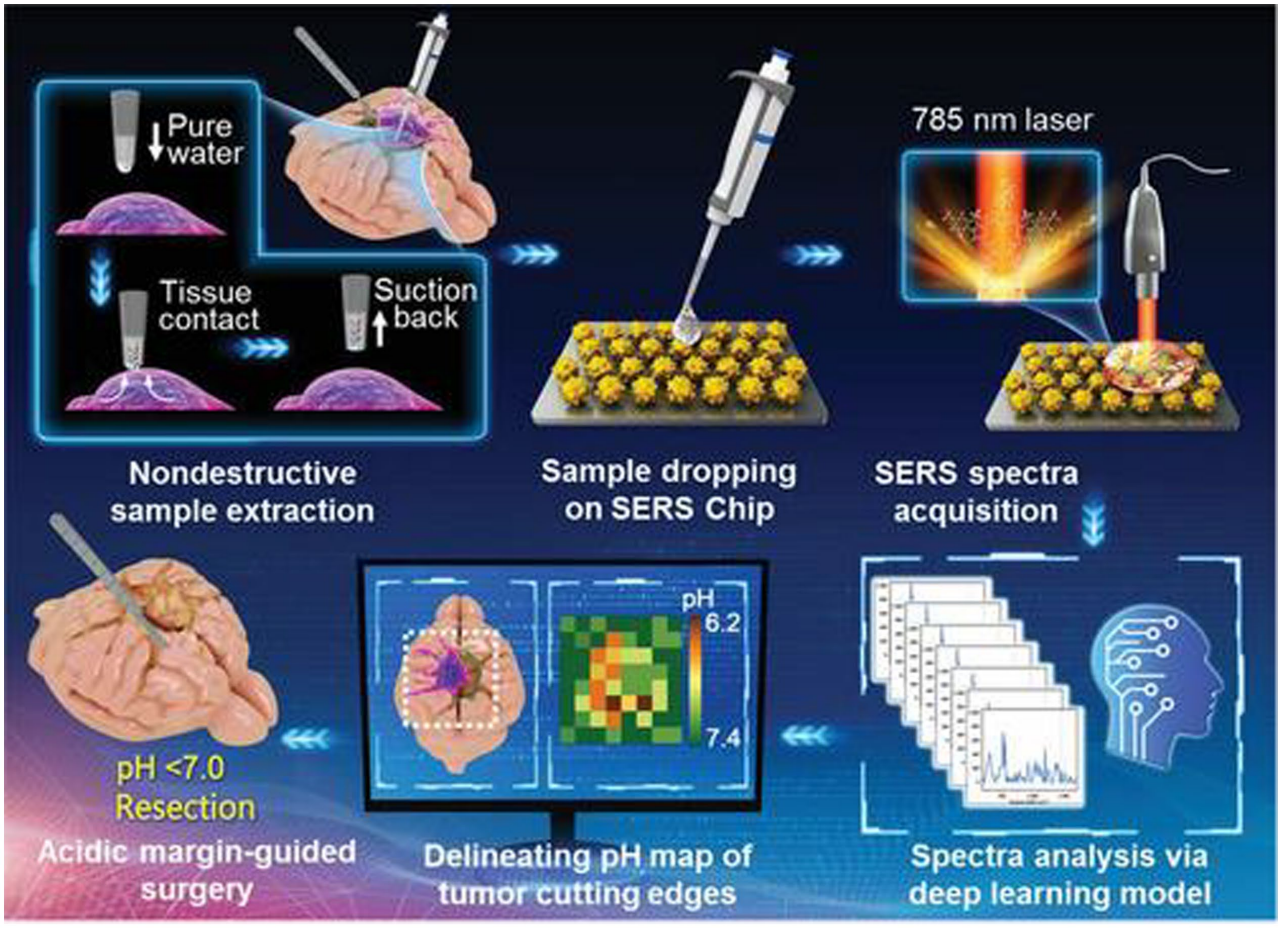
Schematic diagram of the SERS navigation system intraoperatively delineating acidic margin of glioma. A trace amount of pure water (≈0.4 μL) in the pipette tip contacts suspicious tissue at the tumor cutting edge for 2~4 s. Then, the water droplet is sucked back and dripped onto a pH-sensitive SERS chip. The Raman spectra of the aqueous sample on the SERS chip were acquired by a handheld Raman scanner equipped with a 785 nm laser. The pH map of tumor cutting edges was intraoperatively delineated with the assistance of a deep learning model by automatically analyzing the Raman spectra. With the guidance of the pH map, acidic tissues with pH values less than 7.0 were excised (50). Copyright 2022 John Wiley and Sons Ltd.

Li et al. (50) reported an intelligent SERS navigation system for describing the acidic edge of glioma with a nondestructive way (Figure 3). They utilized the water droplet extraction to transfer the acidic microenvironment of the tumor cutting edge into a drop of water. Then, they put the drop on a pH-sensitive SERS chip that had been modified by IR7p, which underwent protonation and deprotonation according to different environments, giving color changes and SERS signal variation. Based on its color sensing method, the acidic range of the environment was determined. The model was applied to a rat tumor-bearing model and the intraoperative resection of glioma. The results showed that the recurrence time of the tumor in the group of rat glioma resection guided by SERS technology was significantly later than that of other groups. Further, they applied this technology to human glioma tissue. It was shown that the detection results of SERS technology guided by pH were consistent with those of hematoxylin–eosin (HE) staining, and it could quickly depict the pH map of the tumor resection bed. Acidity-related cancer cell density and proliferation levels were shown in animal models and tumor margin tissues excised from glioma patients. Compared with conventional strategies used in clinical practice, the overall survival rate of postoperative animal models guided by the SERS system significantly increased. This technology is expected to accelerate the clinical transformation of acidic edge-guided surgery.

**Figure 4:**
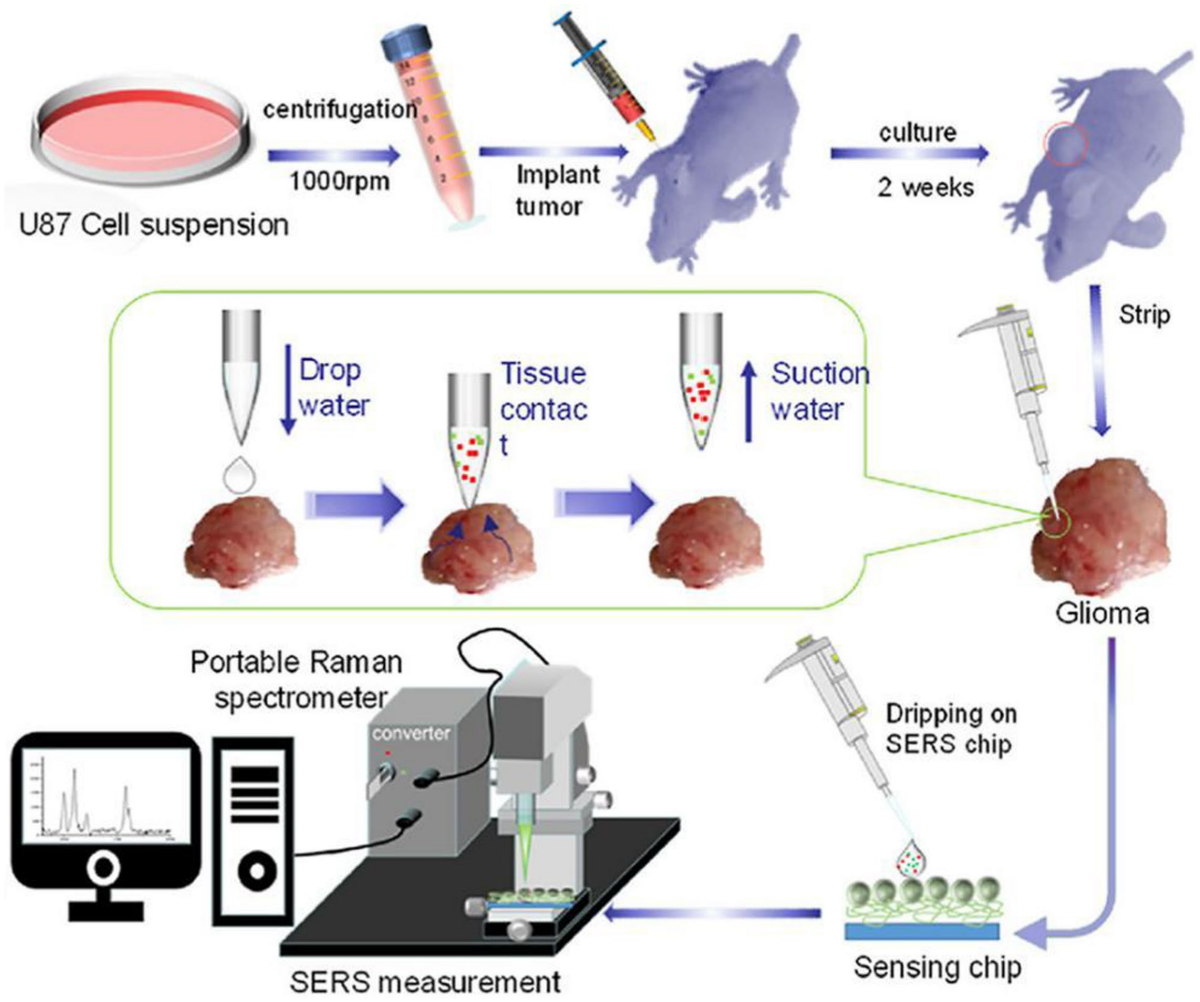
Preparation of a tumor-bearing model in a naked mouse, the extraction of the interstitial fluid of a glioma sample, and its SERS measurement by a portable optical-fiber Raman spectrometer equipped with a 532 nm laser (23). Copyright 2022 Elsevier Science.

Zhang and Xu developed a similar SERS strategy for rapid diagnosis of glioma boundary by using an ultrasensitive SERS substrate and a portable Raman spectrometer (Figure 4). They prepared a SERS substrate by the self-assembled silver nanoparticle monolayer bridged by polyelectrolyte, followed by an assembled layer of 4-MPY. They constructed pure water droplet arrays in different regions of tumor tissue, which can allow the interstitial fluid of tumor tissue to diffuse into water. By monitoring the peak intensity ratio of 4-MPY (1,091 cm^−1^/1,580 cm^−1^) recorded by a portable Raman spectrometer, the acidification characteristics of tumor regions were revealed, which shows a different pH relative to normal tissue, thereby accurately distinguishing the tumor boundaries. The detection results were consistent with the results achieved by the microelectrochemical pH electrode. This method has no harm to the surgical tissue and is expected to replace the rapid pathological detection during the operation of glioma and become a feasible technology for intraoperative navigation (23).

### 3.2 Illuminating glioma in living body by SERS

**Figure 5:**
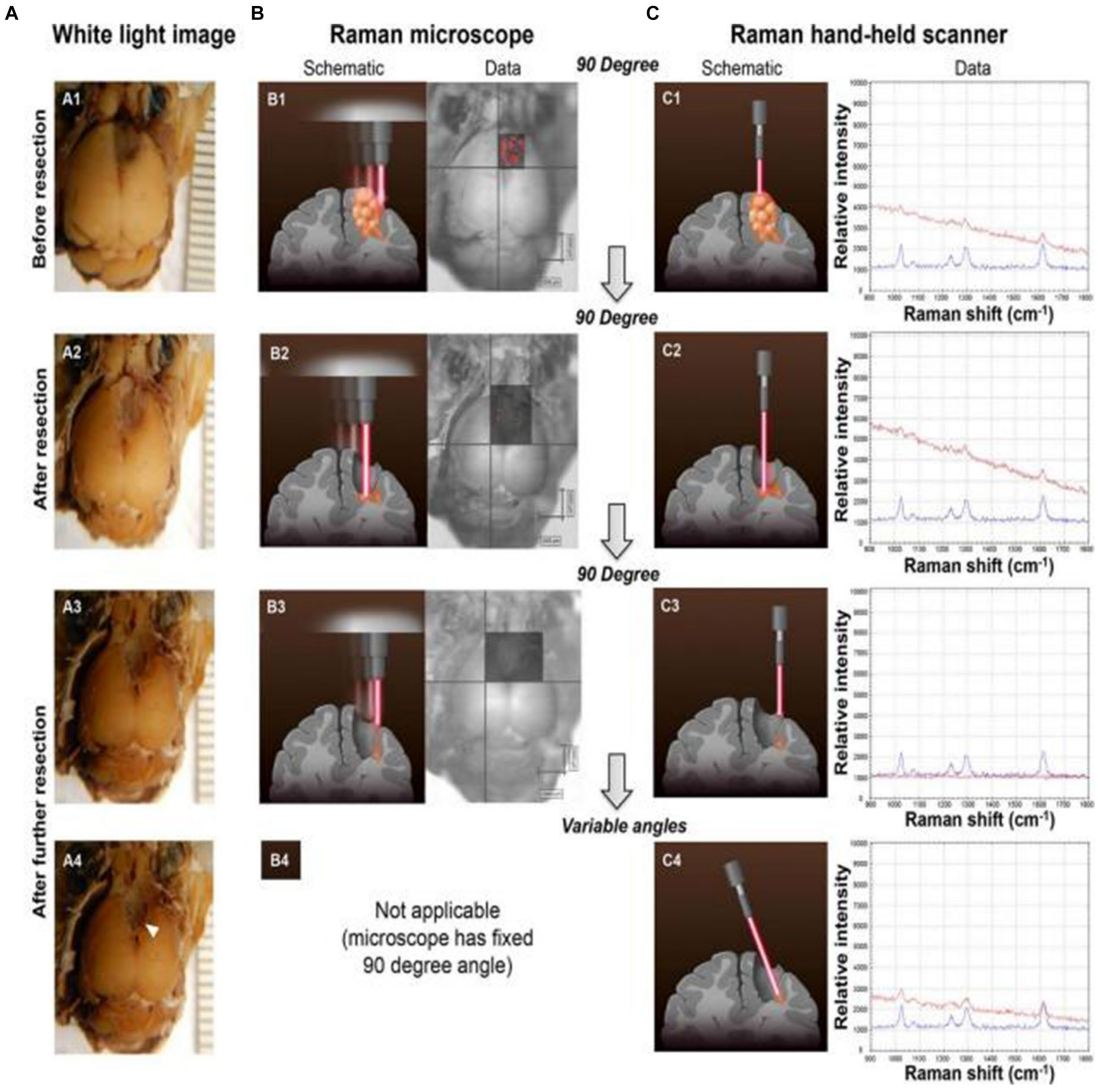
Glioblastoma (GBM) resection with the guidance of a Raman microscope. **(A)** Photographs of the intact brain before **(A1)** and after **(A2,A3)** successive tumor resections guided by the Raman microscope (fixed 90 angle). When the hand-held Raman scanner was used at variable angles after these resection steps, additional microscopic tumor tissue was detected (location depicted by arrowhead in **A4**). **(B)** SERS images acquired with the Raman microscope before **(B1)** and after **(B2,B3)** successive tumor resections. **(C)** The hand-held Raman scanner was used for verification of signal **(C1–C3)** observed with the Raman microscope **(B1–B3)**. **(C4)** Angulated scanning of the lateral wall of the resection bed with the hand-held Raman scanner detected microscopic tumor, which had been missed by the Raman microscope. Tissue was left in place for histological verification *in situ* (red Raman spectra = nanoparticles detected in brain tissue; blue Raman spectra = SERS nanoparticle standard as control) (51). Copyright 2014 ACS.

By the tissue injection of SERS nanoprobes to start circulatory system delivery, SERS technology can determine the boundaries of gliomas in a living body. Karabeber et al. (51) loaded tumors on mice to simulate human glioblastoma. After injecting Au@SiO_2_ nanoparticles through the tail vein and circulating in the body for 24 h, brain tissue was taken and fixed in formaldehyde. Mouse glioma tissue was detected under white light, static Raman instrument, and handheld Raman spectrometer (Figure 5). When glioma tissue was removed, residual nano signals of tumor tissue could be detected using a static Raman instrument at a vertical angle facing the tissue. Once these residual signal tissues were removed, no residual tumor tissue was observed. However, when the angle was changed, residual glioma tissue was still found in the brain tissue around the tumor. Subsequently, after slicing the tissue area and conducting a pathological examination, it was indeed a residual tumor tissue. This study proves that the detection of glioma tissue can be achieved through SERS technology, and the portable Raman analyzer has a more convenient and sensitive detection method.

**Figure 6:**
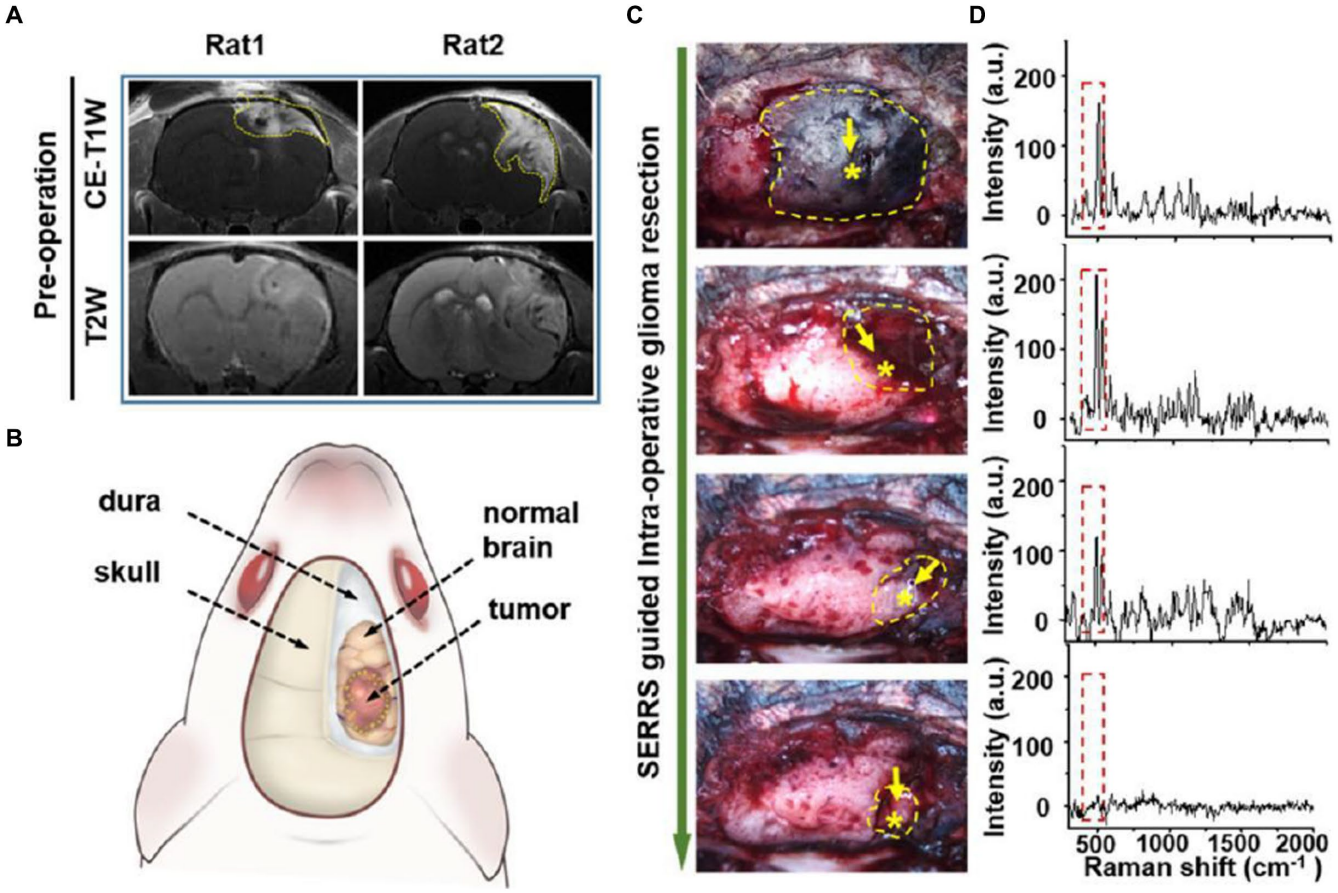
AuS-IR7 intraoperatively guiding glioma resection in live mouse models. **(A)** Pre-operative T1W and T2W MR images of rat brain bearing orthotopic glioma xenograft. Tumor was located in the cortex (left, rat1) or corpus striatum (right, rat2). **(B)** Drawing up a surgical plan before the craniotomy. **(C)** Sequential glioma resection pictures of a rat bearing orthotopic glioblastoma xenograft. Yellow dashes marked the areas with detectable Raman signals. Star symbols present the points with the highest Raman signal. **(D)** *In vivo* Raman spectra at the sequential steps during the SERRS-guided tumor resection. The characteristic twin peaks of AuS-IR7 were highlighted by dash squares (52). Copyright 2019 ACS.

Han et al. (52) developed a AuS-IR7 probe, and they injected it intravenously into mice (Figure 6). After reaching the tumor tissue with the probe, they measured the area with the strongest surface-enhanced resonant Raman scattering (SERRS) of AuS-IR7, guiding the edge resection. After resection, MRI was used to evaluate the postoperative prognosis of the SERS-guided resection in comparison to the white light-guided resection. The MRI images showed that the tumor tissue excised under white light showed an enhanced MRI signal on the fifth day. On the 12th day, the recurrent tumor tissue reached 14 mm^3^, and almost occupied the Cerebral hemisphere 15 days later. Interestingly, the tumor tissue resected under the SERS guide did not exhibit noticeable MRI signal enhancement, proving that SERS-guided resection provided a better prognosis.

Diaz et al. (53) also realized the transmission of SERS-active gold nanoparticles through the blood–brain barrier through focused ultrasound so that nanoparticles can be accurately injected into tumor tissue, and tumor boundaries can be accurately identified through SERS measurement to achieve resection.

### 3.3 SERS techniques in liquid biopsy for early diagnosis of tumors

The preventive measures currently taken for malignant tumor cells in clinical practice are still secondary prevention, namely early detection and early treatment. Early detection and early treatment can achieve maximum relief of patient pain while obtaining the best therapeutic effect. Pathological biopsy is mainly used as the gold standard in clinical practice to determine tumor type and staging (54). Because it is an invasive operation that causes great damage to patients, a new non-invasive method for detecting tumor cells is urgently needed. Some biogenic substances in blood were also used for biomarkers, such as Alpha-fetoprotein (AFP), carcinoembryonic antigen (CEA), carbohydrate antigen 153 (CA153), carbohydrate antigen 199 (CA199), carcinoembryonic antigen 125 (CA125), prostate-specific antigen (PSA), etc. (55, 56). However, these biomarkers are often used for recurrent diagnosis and are not sensitive to early diagnosis.

Liquid biopsy can achieve non-invasive detection while minimizing patient pain. Research shows that endogenous substances in the body can be stored in the internal environment of the human body, such as blood, interstitial fluid, urine, saliva, and cerebrospinal fluid, and these substances can be revealed by SERS. The endogenous substances found in recent years mainly include circulating tumor cells (CTCs), circulating tumor DNA (ctDNA), microRNAs (miRNAs) and some substances secreted in exosomes (57, 58). Especially for glioma tissue, early detection and concurrent surgical resection can preserve the functional area of tissue to the maximum extent, and resect tumor tissue to the maximum extent, which can greatly increase the prognosis and quality of life of patients. Due to the invasive growth of glioma, blood–brain barrier is damaged. So the extracellular vesicles (EVs) of biological fluid that are not easy to appear in the blood. At the same time, the presence of cerebrospinal fluid greatly increases the chances of this substance being present.

**Figure 7:**
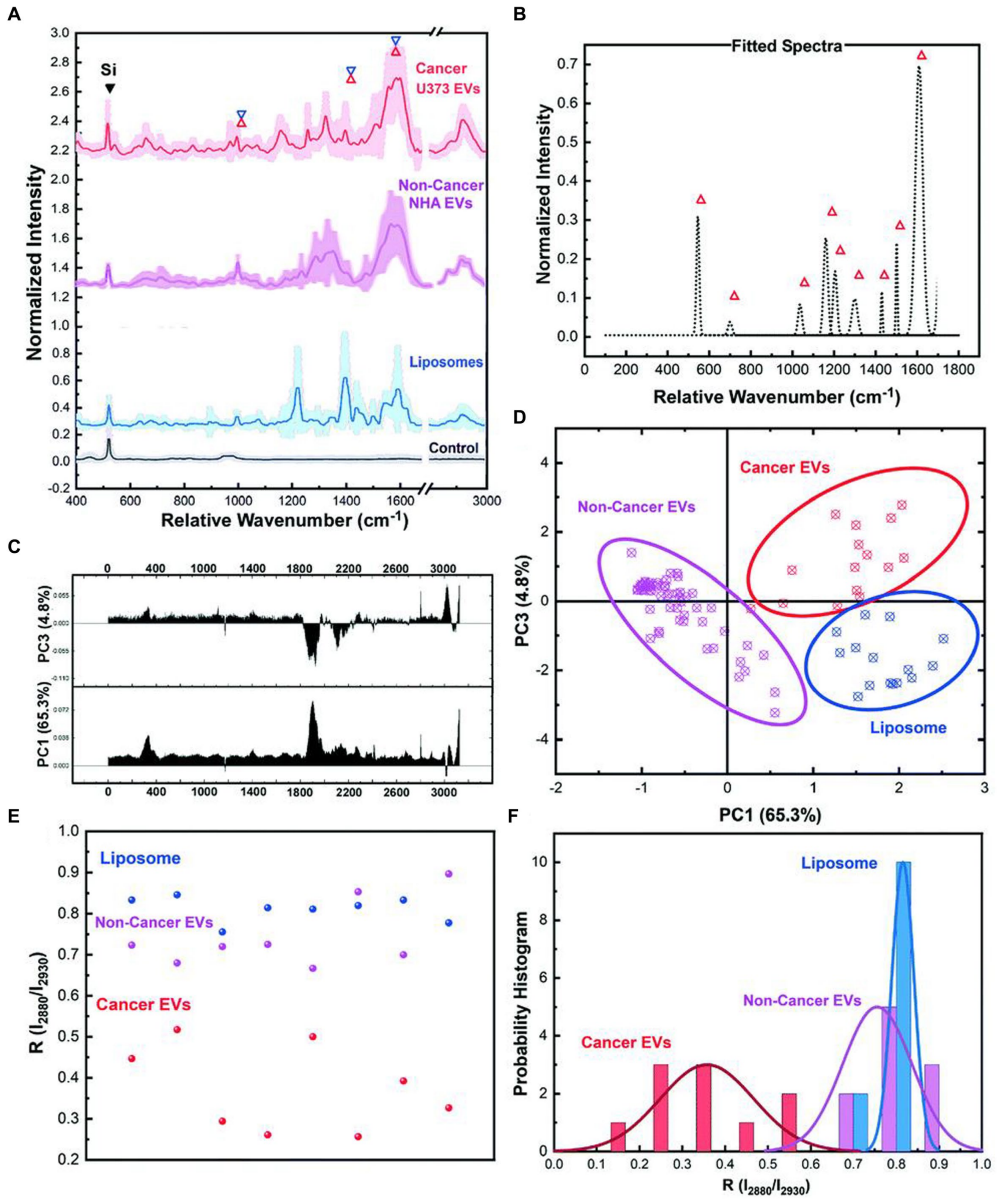
Ultrasensitive SERS detection of EVs from non-cancerous (NHA) and cancerous (U373) glial cells as well as liposomes with the nanobowtie microfluidic chip. **(A)** SERS characterization for investigating the specific Raman scattering signals of EVs derived from non-cancerous glial cells (NHA), cancerous glioma cells (U373) and liposomes. Each spectrum is the mean value of the spectra and the SD is demonstrated with lighter color. For each sample, a minimum of 15 data points were used after the normalization process and elimination of the out of range data points. **(B)** Unique peaks existing in EV spectra that did not appear for liposomes or were considerably weak. **(C)** PC1 and PC2 loading Raman bands based on which the **(D)** PCA score plot of the SERS data, demonstrating the distinct position of the spectra from each sample, and that each type is defined. Each point is related to one experiment. In the same color, the 95% confidence ellipses are demonstrated. **(E)** Comparison analyses of lipid membrane properties (Chol amount) based on the R = I_2,880 cm−1_/I_2,930 cm−1_ intensity ratio distribution. Each point is related to one trial. **(F)** The histogram and correlated fit of R-values for liposomes and EVs, demonstrating the composition of Dioleyl phosphatidylcholine (DOPC): Chol while showing the heterogeneity (59). Copyright 2021 RSC.

Jalal et al. (59) developed a nanorobot-shaped antenna-decorated microfluidic device to identify EVs (Figure 7). They separated EVs from non-cancer cell lines and two different glioma cell lines (U373 and U87) to measure their SERS spectra. The SERS data displayed characteristic peaks layed near 1,250, 1,325, and 1,580 cm^−1^. Liposomes, U373, and U87 can be accurately distinguished by the covariance PCA algorithm. They can be considered as the special fingerprints of U373 and U87 EVs. This study displays that the SERS integrated with microfluidic devices provides the potential for the diagnosis and treatment of glioma.

**Figure 8:**
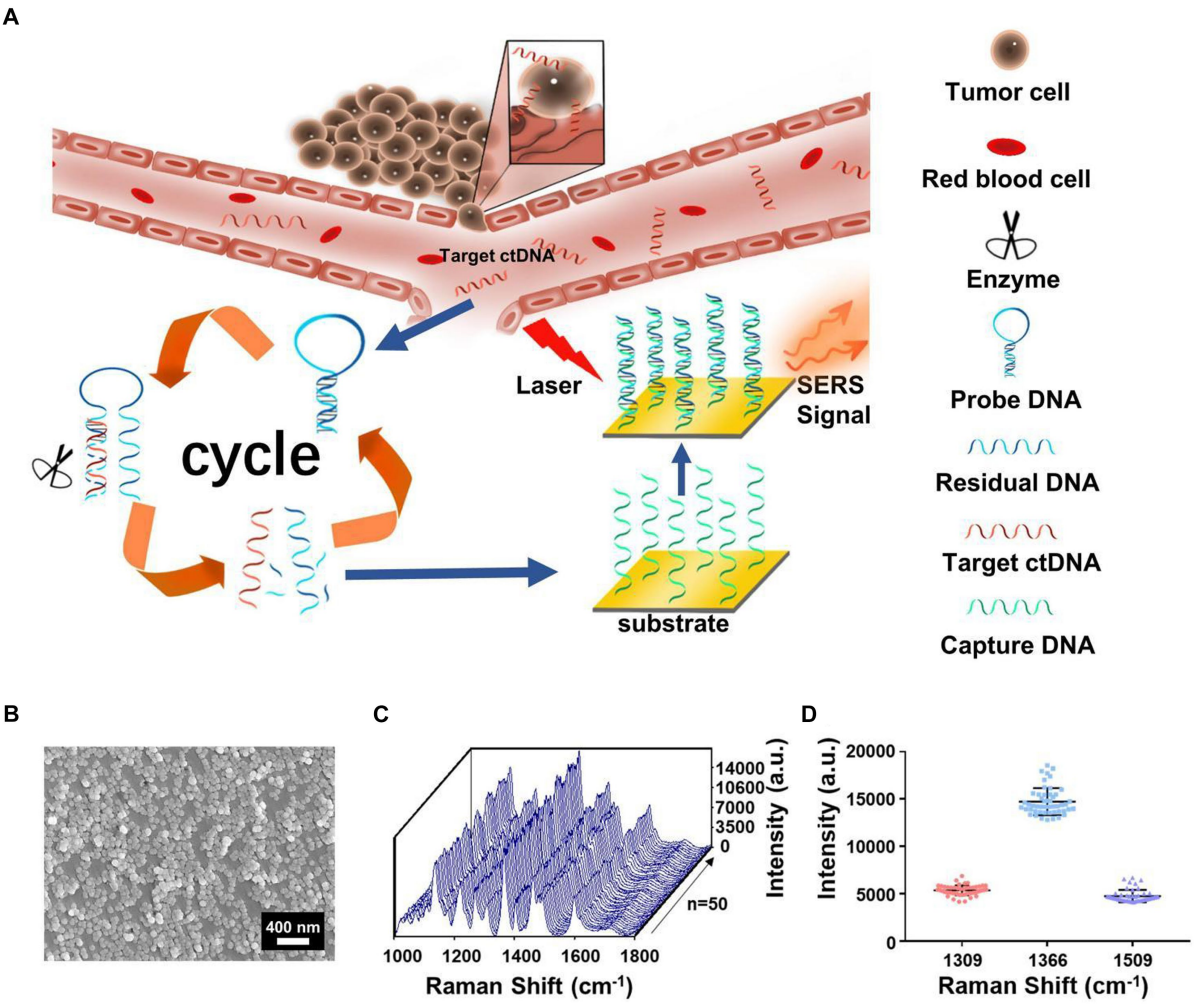
**(A)** The integration of cycled enzymatic DNA cleavage/amplification and SERS for sensitive detection of ctDNA. **(B)** The scanning electron microscopic image of the AuNPs@Si substrate. **(C)** SERS spectra of testing Cy5 collected from 50 random spots on the AuNPs@Si substrate in a single assay. **(D)** Averaged SERS intensities at three peaks from 50 random spots, respectively (60). Copyright 2021 Frontiers Media SA.

In recent years, ctDNA has become an emerging liquid biopsy biomarker, mainly derived from cell apoptosis, necrosis, and secretion processes. The content of single-base mutated ctDNA sequences increased in diffuse intrinsic pontine gliomas (DIPGs). Miao et al. (60) reported a method by combining cyclic enzyme DNA amplification technology and gold nanoparticles@silicon (AuNPs@Si) assisted SERS technique, as shown in Figure 8. They designed an oligonucleotide probe folded into a stem ring hairpin, labeled with a cyanine dye (Cy5) at the 5′ end. The stem ring structure of the oligonucleotide probe could be changed by hybridization with the target sequence of the mutant ctDNA, forming a new double helix structure. In the new double helix structure, the prominent 3′ end can be specifically recognized by the Exo III enzyme and gradually cleaved into nucleotides. After the cleavage process was completed, the target sequence of ctDNA would be released into the solution and recycled for the next round of enzymatic DNA cleavage of oligomer probes. In this way, the residual DNA sequence generated by enzymatic cleavage of oligonucleotide probes accumulated to a large amount through this cyclic reaction. They added AuNPs@Si to hybridize, causing the Cy5 tag closer to the substrate, efficiently generating dense SERS signals. ctDNA can initiate the cyclic generation of residual DNA sequences, thereby achieving SERS detection of ctDNA based on the content of Cy5. The results indicated the SERS intensity at 1,366 cm^−1^ showed a particular linear relationship when the concentration of ctDNA increased from 1 pM to 0 fM. Therefore, an early diagnosis of glioma was achieved based on the changes in SERS intensity displayed by the changes in the content of ctDNA in blood.

### 3.4 Chemical information revealed by label-free SERS

Using a specific spectrum generated by a certain molecular substance to identify the species has become popular in recent years and has grown fast with the progress of deep learning and artificial intelligence (AI) techniques. The label-free SERS can reveal the chemical information of analytes, which supplies richer component information and avoid the false positive results that could happen in SERS-labeling methods. SERS utilizes the molecular resonance hotspot effect to enhance the spectrum of the molecular substance, making it easier to identify the difference between tumor tissue and normal brain tissue.

**Figure 9:**
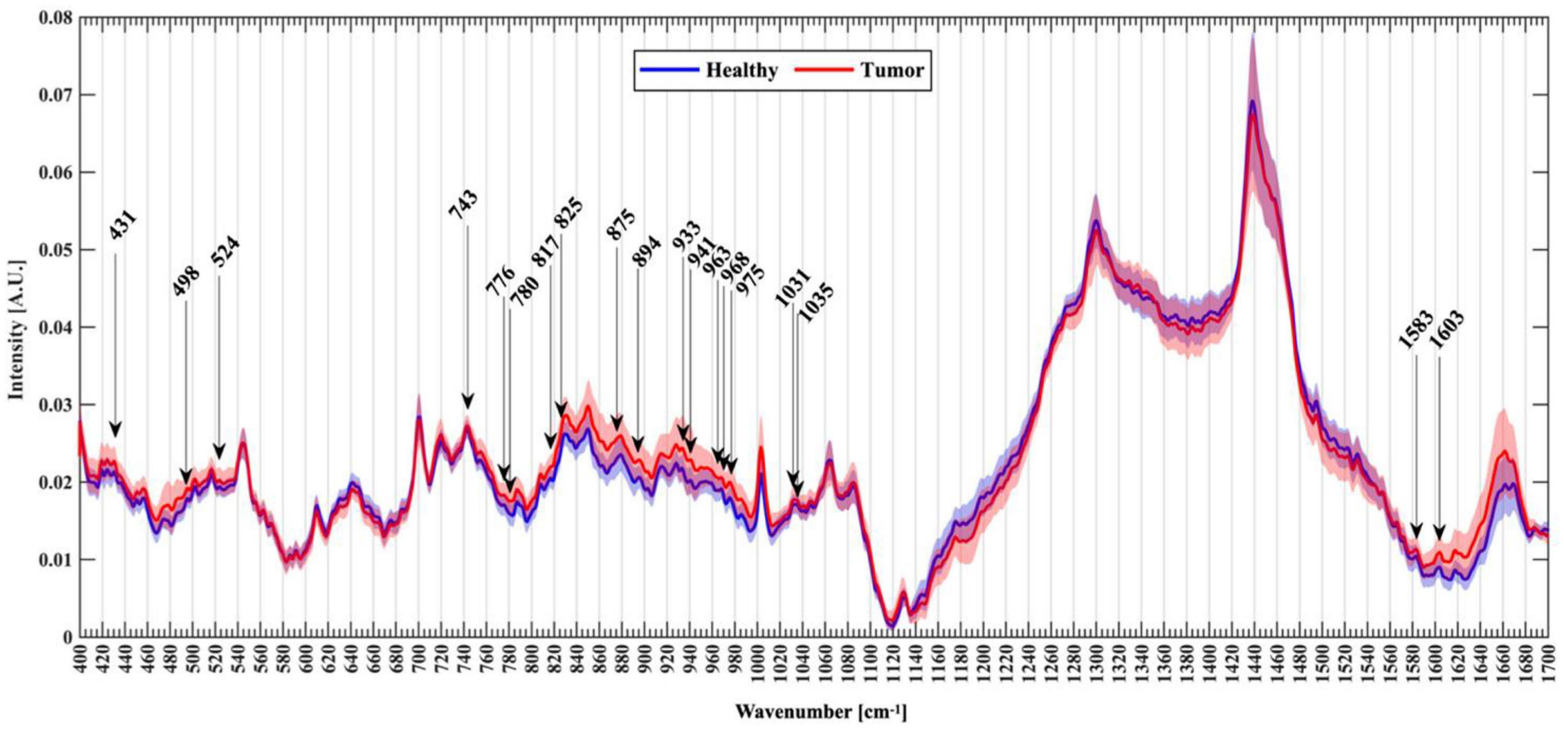
Normalized mean spectra with standard deviation for healthy (blue) and tumor patients (red). Arrows mark the new Raman peaks identified (61). Copyright 2021 MDPI.

Riva et al. (61) analyzed normal and fresh tumor tissues by label-free SERS, and achieved SERS imaging of 63 fresh brain tissues. They collected 3,450 spectra, including 1,377 healthy tissue spectra and 2,073 tumor tissue spectra. Through algorithm recognition, 60 capable characteristic peaks were identified and screened. Based on the spectral analysis, glioma tissue was compared with normal tissue, 19 new characteristic peaks were discovered to be able to distinguish glioma tissue from healthy brain tissue, as shown in Figure 9. They assigned these Raman bands to proteins (524, 933, 963,1,031, 1,035, 1,583, 1,603 cm^−1^), nuclear acids (498, 780, 825, and 894 cm^−1^), lips (431, 776, 875, 968 cm^−1^), collagen (817 cm^−1^), glycogen (941 cm^−1^), theme content (743 cm^−1^), and calibration (975 cm^−1^). This study is helpful for understanding the occurrence and infiltration relationship of glioma.

**Figure 10:**
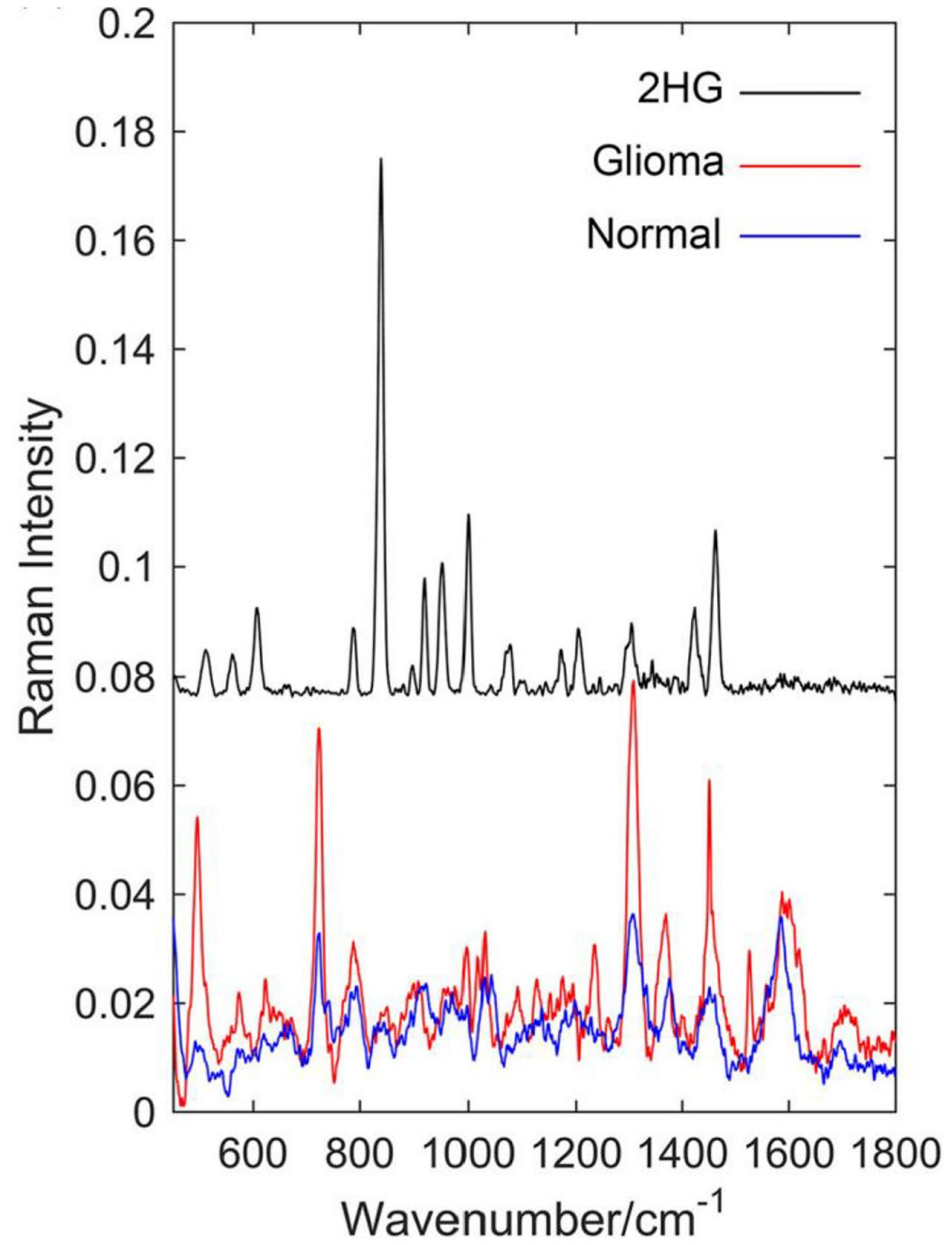
Raman spectrum of solid 2-hydroxyglutarate (2HG), the mean Raman spectrum of 23 glioma supernatant, and the mean Raman spectrum of 24 normal supernatant (62). Copyright 2019 John Wiley and Sons Ltd.

Sun et al. (62) compared the Raman spectra of glioma tissue, normal tissue, and 2-hydroxyglutarate (2HG). They selected 24 normal brain tissue and 23 AC/DC tissue samples (Figure 10). After the biopsy, each tissue was cut into 1 mm-thickness sections, and then physiological saline was added. The supernatant was dripped on a PEGylated SERS substrate and their corresponding SERS spectra were measured. Compared with normal tissue, stronger Raman peaks around 500–800, 1,000, and 1,600 cm^−1^ were found, indicating we enable the differential diagnosis of glioma and normal brain tissue by means of SERS. The summary of the application of SERS in glioma is shown in Table 2.

**Table 2:** Summary of the application of SERS in glioma.

Authors	SERS detection probe	Sample	Function	Mechanism	Refs.
Li et al.	IR7p	Glioma tissue	pH-sensitive	The microenvironment of gliomas is acidic, and the boundary of gliomas can be determined by detecting the pH value of the environment using SERS	(50)
Zhang and Xu et al.	4-MPY	Glioma tissue	pH-sensitive	The microenvironment of gliomas is acidic, and the boundary of gliomas can be determined by detecting the pH value of the environment using SERS	(23)
Han et al.	AuS-IR7	Glioma tissue	Identifying tumor tissue	AuS-IR7 can reach the edge of tumor tissue with blood, and SERS can be used for specific detection of AuS-IR7, thereby determining the tumor edge	(52)
Diaz et al.	AuNPs	Glioma tissue	Identifying tumor tissue	By focused ultrasound to allow gold nanoparticles to enter tumor tissue through the blood–brain barrier, and then detecting the nanoparticles through SERS to achieve detection of tumor tissue	(53)
Jalal et al.	Extracellular vesicles (EVs)	Glioma cell	Tumor specific substances	The use of SERS can detect tumor specific secretions EVs, thereby achieving the detection of tumor cells	(59)
Miao et al.	ctDNA	Glioma cell	Tumor specific substances	When the ctDNA content of tumor tissue significantly increases, its SERS spectrum will show a specific linear relationship at 1366 cm-1	(60)
Riva et al.	Free	Glioma tissue	-	Normal and fresh tumor tissues were analyzed using unlabeled SERS. Based on spectral analysis, glioma tissue was compared with normal tissue and 19 new characteristic peaks were found, which can distinguish glioma from healthy brain tissue	(61)
Sun et al.	Free	Glioma tissue	-	By using SERS to detect glioma and normal brain tissue, stronger Raman peaks were found in glioma tissue around 500–800, 1,000, and 1,600 cm-1 compared to normal tissue	(62)

## 4 Summary and outlook

Since the birth of SERS technology, it has been widely used for detecting biological samples with its high sensitivity, specificity, non-invasive, and efficiency. This article briefly introduces the structure and responsive mechanisms of SERS nanoprobes and reviews their applications in glioma-related studies, including the detection of tumor cell microenvironment using SERS technology, SERS imaging of glioma tissue in living body, SERS technology for glioma-related liquid biopsy, and the application of free-label SERS technology to detect fresh glioma specimens for qualitative diagnosis of glioma.

**Figure 11:**
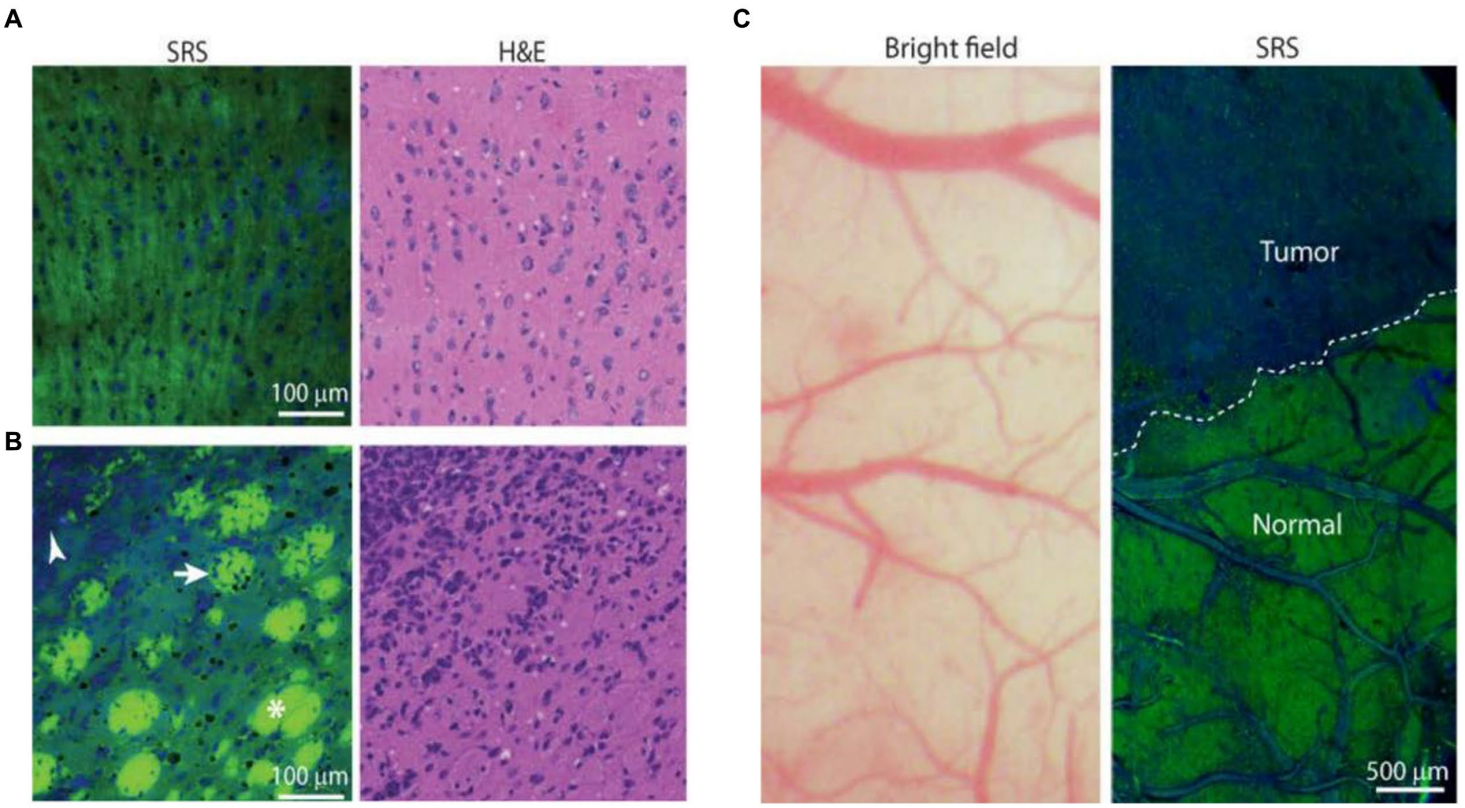
SRS images of frozen human GBM xenograft. **(A)** High-magnification view of normal to minimally hypercellular cortex. **(B)** Infiltrating glioma with normal white matter bundles (asterisk), tumor-infiltrated bundles (arrow), and dense tumor cells (arrowhead). **(C)** Bright-field microscopy appears grossly normal, whereas SRS microscopy within the same field of view demonstrates distinctions between tumor-infiltrated areas and non-infiltrated brain (normal), with a normal brain–tumor interface (dashed line) (66). Copyright 2013 American Association for the Advancement of Science.

These studies provide broad prospects for the application of SERS technology in biomedicine. However, it is undeniable that SERS technology also has many shortcomings that hinder its application in clinical medicine. Firstly, sample collection. Most Raman detection devices require the transfer of intraoperative tissue to the detection equipment for detection. During the transfer process, it is difficult to avoid causing tissue sample denaturation, which affects signal changes. At the same time, during SERS measurement, the signal is highly susceptible to the influence of the surrounding environment and probe concentration (63). Secondly, more convincing results on the long-term tracking report on the cytotoxicity generated by the injection of nanoparticles into veins are needed. If applied to human tissues, long-term uncertainty and biocompatibility may arise. For the commonly used SERS tags, less toxicity to the human body, safe and unambiguous metabolic pathway, and more obvious signal enhancement (64, 65). Thirdly, due to the need for SERS technology to collect a large amount of spectral information and preprocess the spectral information, the large sample size and complex program are unachievable in clinic. Therefore, it is necessary to explore a fast method for identifying and analyzing spectral information to simplify the complex program required for spectral processing. The development of high-speed imaging technology may be a good solution. Coherent anti-Stokes Raman scattering (CARS) and stimulated Raman scattering (SRS) have been rapidly developed in the last two decades. CARS can detect lipid content. Evans et al. (67) used a CARS microscope to detect the lipid content, and they observed a significant decrease in tumor tissue signal, proving that CARS technology can detect tumor cell boundaries. SRS microspectral technology can generate different signals based on different protein and lipid contents, and can also display different regions. Ji et al. (66) implanted glioblastoma cells into mice, allowing them to infiltrate and grow into tumors. The slices were then subjected to SRS imaging, and the spectral information generated accurately distinguished the protein-rich tumor infiltrating areas from normal brain tissue, indicating that SRS technology is a promising technology in clinical practice (Figure 11). All this indicates that with the help of various effective Raman approaches, people are constantly advancing their understanding of brain glioma. We believe that in the near future, these spectral technologies will continue to carry forward and serve biomedicine deeply.

## Author contributions

GY: Writing – original draft. KZ: Writing – review & editing. WX: Writing – review & editing. SX: Writing – review & editing.

## References

[1] Omuro A LM DA. Glioblastoma and other malignant gliomas. JAMA. (2013) 310:1842–50. doi: 10.1001/jama.2013.280319 24193082

[2] Sarkar S Yong VW. Inflammatory cytokine modulation of matrix metalloproteinase expression and invasiveness of glioma cells in a 3-dimensional collagen matrix. J Neuro-Oncol. (2009) 91:157–64. doi: 10.1007/s11060-008-9695-1, PMID: 18802741

[3] Pavlova N Thompson CB. The emerging hallmarks of cancer metabolism. Cell Metab. (2016) 23:27–47. doi: 10.1016/j.cmet.2015.12.006, PMID: 26771115 PMC4715268

[4] Reinfeld B Madden MZ Wolf MM Chytil A Bader JE Patterson AR. Cell-programmed nutrient partitioning in the tumour microenvironment. Nature. (2021) 593:282–8. doi: 10.1038/s41586-021-03442-1, PMID: 33828302 PMC8122068

[5] Hanahan D Weinberg RA. Hallmarks of cancer: the next generation. Cell. (2011) 144:646–74. doi: 10.1016/j.cell.2011.02.013 21376230

[6] Wong K Young GS Makale M Hu X Yildirim N Cui K. Characterization of a human tumorsphere glioma orthotopic model using magnetic resonance imaging. J Neuro-Oncol. (2011) 104:473–81. doi: 10.1007/s11060-010-0517-x, PMID: 21240539 PMC3161186

[7] Hou Y Tang J. Advantages of using 3D intraoperative ultrasound and intraoperative MRI in glioma surgery. Front Oncol. (2022) 12:925371. doi: 10.3389/fonc.2022.925371, PMID: 35719958 PMC9203997

[8] Bloch O Garcia A Anbunesan SN Frusciante R Bec J Marcu L. FLGS-04 Fluorescence lifetime imaging (FLIM) is a dye-free, high sensitivity approach for fluorescence guided surgery in high-grade and low-grade gliomas. Neuro-Oncology. (2021) 23:226. doi: 10.1093/neuonc/noab196.90832822486

[9] Xu H Wang H Xu Z Bian S Xu Z Zhang H. The multifaceted roles of peptides in “always-on” near-infrared fluorescent probes for tumor imaging. Bioorg Chem. (2022) 129:106182. doi: 10.1016/j.bioorg.2022.106182, PMID: 36341739

[10] Andrea B Della Puppa A. 5-ALA fluorescence on tumors different from malignant gliomas. Review of the literature and our experience. J Neurosurg Sci. (2019) 63:661–9. doi: 10.23736/S0390-5616.19.04766-0 31355622

[11] Zhang N Shang Z Wang Z Meng X Li Z Tian H. Molecular pathological expression in malignant gliomas resected by fluorescein sodium-guiding under the yellow 560 nm surgical microscope filter. World J Surg Oncol. (2018) 16:195. doi: 10.1186/s12957-018-1495-2, PMID: 30285781 PMC6167783

[12] Jia Y Wang X Hu D Wang P Liu Q Zhang X. Phototheranostics: active targeting of orthotopic glioma using biomimetic proteolipid nanoparticles. ACS Nano. (2019) 13:386–98. doi: 10.1021/acsnano.8b06556 30576599

[13] Jaffrey S. RNA-based fluorescent biosensors for detecting metabolites in vitro and in living cells. Adv Pharmacol. (2018) 82:187–203. doi: 10.1016/bs.apha.2017.09.005, PMID: 29413520

[14] Zhang C Wei ZH Ye BC. Imaging and tracing of intracellular metabolites utilizing genetically encoded fluorescent biosensors. Biotechnol J. (2013) 8:1280–91. doi: 10.1002/biot.201300001, PMID: 24591186

[15] Duffau H. The necessity of preserving brain functions in glioma surgery: the crucial role of intraoperative awake mapping. World Neurosurg. (2011) 76:525–7. doi: 10.1016/j.wneu.2011.07.040, PMID: 22251497

[16] Jones R Hooper DC Zhang L Wolverson D Valev VK. Raman techniques: fundamentals and frontiers. Nanoscale Res Lett. (2019) 14:231. doi: 10.1186/s11671-019-3039-2 31300945 PMC6626094

[17] Krafft C Schmitt M Schie IW Cialla-May D Matthäus C Bocklitz T. Label-free molecular imaging of biological cells and tissues by linear and nonlinear Raman spectroscopic approaches. Angew Chem Int Ed Engl. (2017) 56:4392–430. doi: 10.1002/anie.201607604 27862751

[18] Li M Xu J Romero-Gonzalez M Banwart SA Huang WE. Single cell Raman spectroscopy for cell sorting and imaging. Opin Biotechnol. (2012) 23:56–63. doi: 10.1016/j.copbio.2011.11.019 22138495

[19] Gandra N Singamaneni S. Bilayered Raman-intense gold nanostructures with hidden tags (BRIGHTs) for high-resolution bioimaging. Adv Mater. (2013) 25:1022–7. doi: 10.1002/adma.201203415 23161698

[20] Fleischmann M Hendra PJ McQuillan AJ. Raman spectra of pyridine adsorbed at a silver electrode. Chem Phys Lett. (1974) 26:163–6. doi: 10.1016/0009-2614(74)85388-1

[21] Lane L Qian X Nie S. SERS nanoparticles in medicine: from label-free detection to spectroscopic tagging. Chem Rev. (2015) 115:10489–529. doi: 10.1021/acs.chemrev.5b00265, PMID: 26313254

[22] Aleksandra J Jamieson LE Malek K Campbell CJ Choo J Chlopicki S. SERS-based monitoring of the intracellular pH in endothelial cells: the influence of the extracellular environment and tumour necrosis factor-alpha. Analyst. (2015) 140:2321–9. doi: 10.1039/c4an01988a 25485622

[23] Yang G Zhang K Qu X Xu W Xu S. Ratiometric pH-responsive SERS strategy for glioma boundary determination. Talanta. (2022) 250:123750. doi: 10.1016/j.talanta.2022.123750, PMID: 35930977

[24] Zhang C Yao J. Research progress in magnetic immunoassay based on surface enhanced Raman spectroscopy. Chin J Light Scatter. (2023) 35:84–96. doi: 10.13883/j.issn1004-5929.202302001

[25] He C Xu L Lin M Lin D Chen Y Xu Y. Research progress on surface-enhanced Raman spectroscopy liquid biopsy technology. Chin J Light Scatter. (2023) 35:160–73. doi: 10.13883/j.issn1004-5929.202302008

[26] Wang P Zhu L Zhao B. SPR characteristics of semiconductor nanomaterials and their applications in SERS. Chin J Light Scatter. (2023) 35:150–9. doi: 10.13883/j.issn1004-5929.202302007

[27] Guicheteau J Christesen S Emge D Wilcox P Fountain AW III. Assessing metal nanofabricated substrates for surface-enhanced Raman scattering (SERS) activity and reproducibility. Appl Spectrosc. (2011) 65:144–51. doi: 10.1366/10-06080

[28] Wu J Liang D Jin Q Liu J Zheng M Duan X. Bioorthogonal SERS nanoprobes for mulitplex spectroscopic detection, tumor cell targeting, and tissue imaging. Chem Eur J. (2015) 21:12914–8. doi: 10.1002/chem.201501942, PMID: 26222682

[29] Kun Chen Heyou Han Zhihui Luo. Streptococcus suis II immunoassay based on thorny gold nano particles and surface enhanced Raman scattering. ANALYST 5 (2012) 137, 1259–1264. doi: 10.1039/C2AN15997J 22282767

[30] Han X Ji W Zhao B Ozaki Y. Semiconductor-enhanced Raman scattering: active nanomaterials and applications. Nanoscale. (2017) 9:4847–61. doi: 10.1039/C6NR08693D, PMID: 28150834

[31] Xiang S Zhang L Gao ST Zhao LB. Simulating pH-dependent surface-enhanced Raman spectra by density functional theory calculations. J Raman Spectrosc. (2019) 50:1065–73. doi: 10.1002/jrs.5613

[32] Fang S Huang X Zeng Q Wang L. Metallic nanocrystallites-incorporated ordered mesoporous carbon as labels for a sensitive simultaneous multianalyte electrochemical immunoassay. Biosens Bioelectron. (2015) 73:71–8. doi: 10.1016/j.bios.2015.05.046, PMID: 26046316

[33] Yu H Xia N Liu ZF. SERS titration of 4-Mercaptopyridine self-assembled monolayers at aqueous buffer/gold interfaces. Anal Chem. (1999) 71:1354–8. doi: 10.1021/ac981131+, PMID: 21662958

[34] Hu J Zhao B Xu W Li B Fan Y. Surface-enhanced Raman spectroscopy study on the structure changes of 4-mercaptopyridine adsorbed on silver substrates and silver colloids. Spectrochim Acta A. (2002) 58:2827–34. doi: 10.1016/S1386-1425(02)00074-4 12477026

[35] Bishnoi S Rozell CJ Levin CS Gheith MK Johnson BR Johnson DH. All-optical nanoscale pH meter. Nano Lett. (2006) 6:1687–92. doi: 10.1021/nl060865w, PMID: 16895357

[36] Schwartzberg A Oshiro TY Zhang JZ Huser T Talley CE. Improving nanoprobes using surface-enhanced Raman scattering from 30-nm hollow gold particles. Anal Chem. (2006) 78:4732–6. doi: 10.1021/ac060220g, PMID: 16808490

[37] Ji W Spegazzini N Kitahama Y Chen Y Zhao B Ozaki Y. pH-response mechanism of p-aminobenzenethiol on ag nanoparticles revealed by two-dimensional correlation surface-enhanced Raman scattering spectroscopy. J Phys Chem Lett. (2012) 3:3204–9. doi: 10.1021/jz301428e, PMID: 26296030

[38] Piotrowski P Wrzosek B Królikowska A Bukowska J. A SERS-based pH sensor utilizing 3-amino-5-mercapto-1,2,4-triazole functionalized ag nanoparticles. Analyst. (2014) 139:1101–11. doi: 10.1039/c3an01197f, PMID: 24409451

[39] Guo H Huang Q Leng W Zhan Y Behkam B Willner MR. Bromide ion-functionalized nanoprobes for sensitive and reliable pH measurement by surface-enhanced Raman spectroscopy. Analyst. (2019) 144:7326–35. doi: 10.1039/C9AN01699F, PMID: 31663525

[40] Ji Y Wang W Li X Chen Y Ding C. Enhanced chemiluminescence detection of glutathione based on isoluminol-PSM nanoparticles probe. Talanta. (2016) 150:666–70. doi: 10.1016/j.talanta.2016.01.004, PMID: 26838457

[41] Li J Huang YF Ding Y Yang ZL Li SB Zhou XS. Shell-isolated nanoparticle-enhanced Raman spectroscopy. Nature. (2010) 464:410. doi: 10.1038/nature08907 20237566

[42] Qian X Peng XH Ansari DO Yin-Goen Q Chen GZ Shin DM. In vivo tumor targeting and spectroscopic detection with surface-enhanced Raman nanoparticle tags. Nat Biotechnol. (2008) 26:83–90. doi: 10.1038/nbt1377, PMID: 18157119

[43] Xiao F Zou YX Wang SS Zheng MJ Hu XX Liang H. Modulating the morphology of gold graphitic nanocapsules for plasmon resonance-enhanced multimodal imaging. Anal Chem. (2016) 88:5385–91. doi: 10.1021/acs.analchem.6b00714 27089383

[44] Li J Huang YF Ding Y Yang ZL Li SB Zhou XS. Shell-isolated nanoparticle-enhanced Raman spectroscopy. Nature. (2010) 464:410. 10.1038/nature08907 20237566

[45] Mulvaney S Musick MD Keating CD Natan MJ. Glass-coated, analyte-tagged nanoparticles: a new tagging system based on detection with surface-enhanced Raman scattering. Langmuir. (2003) 19:4784–90. doi: 10.1021/la026706j

[46] Shang W Zeng C du Y Hui H Liang X Chi C. Core–shell gold nanorod@metal–organic framework nanoprobes for multimodality diagnosis of glioma. Adv Mater. (2017) 29:1604381. doi: 10.1002/adma.201604381 27859713

[47] Kircher M de la Zerda A Jokerst JV Zavaleta CL Kempen PJ Mittra E. A brain tumor molecular imaging strategy using a new triple-modality MRI-photoacoustic-Raman nanoparticle. Nat Med. (2012) 18:829–34. doi: 10.1038/nm.2721, PMID: 22504484 PMC3422133

[48] Vendrell M Maiti KK Dhaliwal K Chang YT. Surface-enhanced Raman scattering in cancer detection and imaging. Trends Biotechnol. (2013) 31:249–57. doi: 10.1016/j.tibtech.2013.01.013 23416096

[49] Bhujwalla Z Artemov D Ballesteros P Cerdan S Gillies RJ Solaiyappan M. Combined vascular and extracellular pH imaging of solid tumors. NMR Biomed. (2002) 15:114–9. doi: 10.1002/nbm.743, PMID: 11870907

[50] Jin Z Yue Q Duan W Sui A Zhao B Deng Y. Intelligent SERS navigation system guiding brain tumor surgery by intraoperatively delineating the metabolic acidosis. Adv Sci. (2022) 9:2104935. doi: 10.1002/advs.202104935 PMC8895125 35023300

[51] Karabeber H Huang R Iacono P Samii JM Pitter K Holland EC. Guiding brain tumor resection using surface-enhanced Raman scattering nanoparticles and a hand-held Raman scanner. ACS Nano. (2014) 8:9755–66. doi: 10.1021/nn503948b 25093240 PMC4212801

[52] Han L Duan W Li X Wang C Jin Z Zhai Y. Surface-enhanced resonance Raman scattering-guided brain tumor surgery showing prognostic benefit in rat models. ACS Appl Meter Interf. (2019) 11:15241–50. doi: 10.1021/acsami.9b00227, PMID: 30896915

[53] Diaz R PZ MV O'Reilly MA Burrell K Bebenek M Smith C. Focused ultrasound delivery of Raman nanoparticles across the blood-brain barrier: potential for targeting experimental brain tumors. Nanomed Nanotechnol. (2014) 10:1075–87. doi: 10.1016/j.nano.2013.12.006 PMC4074278 24374363

[54] Xu Y Jia Z Wang LB Ai Y Zhang F Lai M. Large scale tissue histopathology image classification, segmentation, and visualization via deep convolutional activation features. BMC Bioinformatics. (2017) 18:281. doi: 10.1186/s12859-017-1685-x, PMID: 28549410 PMC5446756

[55] Kannan V Vasudevan DM. Organ specific tumor markers: what’s new? Indian J Clin Biochem. (2012) 27:110–20. doi: 10.1007/s12291-011-0173-8 23542399 PMC3358375

[56] Hideaki S Noie T Ohashi M Oba K Takahashi Y. Clinical significance of serum tumor markers for gastric cancer: a systematic review of literature by the task force of the Japanese gastric cancer association. Gastric Cancer. (2014) 12:26–33. doi: 10.1007/s10120-013-0259-5 23572188

[57] Li W Liu JB Hou LK Yu F Zhang J Wu W. Liquid biopsy in lung cancer: significance in diagnostics, prediction, and treatment monitoring. Mol Cancer. (2022) 21:25. doi: 10.1186/s12943-022-01505-z 35057806 PMC8772097

[58] Gul-e-Saba C Akim AM Safdar N Yasmin A Begum S Sung YY. Cancer and disease diagnosis-biosensor as potential diagnostic tool for biomarker detection. J Adv Pharm Technol. (2022) 13:243–7. doi: 10.4103/japtr.japtr_106_22 PMC9784037 36568055

[59] Jalali M Isaac Hosseini I AbdelFatah T Montermini L Wachsmann Hogiu S Rak J. Plasmonic nanobowtiefluidic device for sensitive detection of glioma extracellular vesicles by Raman spectrometry. Lab Chip. (2021) 21:855–66. doi: 10.1039/D0LC00957A, PMID: 33514986

[60] Miao X Fang Q Xiao X Liu S Wu R Yan J. Integrating cycled enzymatic DNA amplification and surface-enhanced Raman scattering for sensitive detection of circulating tumor DNA. Front Mol Biosci. (2021) 8:676065. doi: 10.3389/fmolb.2021.676065, PMID: 34017856 PMC8129026

[61] Riva M Sciortino T Secoli R D’Amico E Moccia S Fernandes B. Glioma biopsies classification using Raman spectroscopy and machine learning models on fresh tissue samples. Cancers. (2021) 13:1073. doi: 10.3390/cancers13051073, PMID: 33802369 PMC7959285

[62] Sun J Fang H Zhang Z Chen M Tian J Chen L. Detection of glioma by surface-enhanced Raman scattering spectra with optimized mathematical methods. J Raman Spectrosc. (2019) 50:1130–40. doi: 10.1002/jrs.5634

[63] Kahraman M Mullen ER Korkmaz A Wachsmann-Hogiu S. Fundamentals and applications of SERS-based bioanalytical sensing. Nanophotonics. (2017) 6:831–52. doi: 10.1515/nanoph-2016-0174

[64] Pratim P Kumar GV. Single-molecule surface-enhanced Raman scattering sensitivity of ag-Core au-shell nanoparticles: revealed by bi-analyte method. J Phys Chem Lett. (2013) 4:1167–71. doi: 10.1021/jz400496n 26282037

[65] Li C Chen S Zhang K. Effect of toxicity of ag nanoparticles on SERS spectral variance of bacteria. Spectrochim Acta A. (2014) 137:1061–6. doi: 10.1016/j.saa.2014.08.155 25291503

[66] Ji M Orringer DA Freudiger CW Ramkissoon S Liu X Lau D. Rapid, label-free detection of brain tumors with stimulated Raman scattering microscopy. Sci Transl Med. (2013) 2:201ra119. doi: 10.1126/scitranslmed.3005954 PMC3806096 24005159

[67] Evans C Xu X Kesari S Xie XS Wong STC Young GS. Chemically-selective imaging of brain structures with CARS microscopy. Opt Express. (2007) 15:12076–87. doi: 10.1364/OE.15.012076, PMID: 19547572

